# Nutraceutical Content and Genetic Diversity Share a Common Pattern in New Pomegranate Genotypes

**DOI:** 10.3390/molecules27020389

**Published:** 2022-01-08

**Authors:** Carmen Arlotta, Valeria Toscano, Claudia Genovese, Pietro Calderaro, Giuseppe Diego Puglia, Salvatore Antonino Raccuia

**Affiliations:** Institute for Agricultural and Forest Systems in the Mediterranean, National Research Council (ISAFOM-CNR), 95128 Catania, Italy; arlottacarmen@gmail.com (C.A.); valeria.toscano@cnr.it (V.T.); pietro.calderaro@cnr.it (P.C.); giuseppediego.puglia@cnr.it (G.D.P.); salvatoreantonino.raccuia@cnr.it (S.A.R.)

**Keywords:** *Punica granatum* L., bio-agronomic traits, genetic markers, antioxidant activity, total phenolic content, carbohydrates, HPAEC-PAD, minerals, potassium, nutraceutical value

## Abstract

The nutraceutical value of pomegranate in the treatment of many diseases is well-documented and is linked to its richness in phenolic compounds. This study aims to evaluate the nutraceutical and genetic diversity of novel pomegranate genotypes (G1–G5) in comparison to leading commercial pomegranate varieties, i.e., ‘Wonderful’, ‘Primosole’, ‘Dente di Cavallo’ and ‘Valenciana’. Morphometric measurements were carried out on fruits, accompanied by chemical characterization (total phenolic content, antioxidant activity, carbohydrates and minerals) and the development of four new polymorphic SSR markers involved in the flavonoid pathway. The cultivars displayed a marked variability in the weight and shape of the fruits, as well as in the weight of the arils and juice yield. The highest level of total phenolic content and antioxidant activity was found in ‘Wonderful’ and G4, while the lowest was in ‘Dente di Cavallo’. Furthermore, the results showed that pomegranate juice is an excellent source of minerals, especially potassium, which plays a key role in organ functioning. The new flavonoid-related markers effectively differentiated the cultivars with the same diversity pattern as morpho-chemical characterization, so the SSRs developed in the present study can be used as a rapid tool for the identification of pomegranate cultivars with relevant nutraceutical traits, such as the new genotypes investigated.

## 1. Introduction

*Punica granatum* L. belongs to the *Punicaceae* family, and it is an ancient and appreciated fruit crop. Pomegranate is a fruit tree widely grown in several countries, especially those with Mediterranean-like climates, characterized by high exposure to sunlight, mild winters with minimal temperatures not lower than −12 °C and dry, hot summers without rain during the last stages of the fruit development. Under such conditions, the fruit can develop to its best size and with an optimal colour and sugar accumulation without the danger of splitting [1]. Pomegranates have traditionally been used for the production of fresh juice from the arils, the edible parts of the fruit, but, recently, there has been a great and increasing demand for industrial processing to obtain bottled juice, jams, oil, supplements and anti-ageing creams [2,3]. The juice represents, on average, 30–40% of the total fruit weight and is a good source of minerals (potassium, phosphorus, calcium, iron and magnesium), as well as glucose, fructose [4] and fibres. Moreover, pomegranate juice contains organic acids (citric acid, malic acid), vitamin C, vitamin E, coenzyme Q and polyphenols, such as ellagitannins (punicalagin) and anthocyanins [5]. The juice concentration, the content of sugars, the colour, the polyphenol content and the quality of the product depend on the variety and degree of ripeness of the fruits. Climatic and environmental conditions can influence these traits. Pomegranate varieties differ in their taste, ranging from sweet to sour, and this is related to the phenols, organic acids and sugars contained in the fruit. The most accepted varieties are those that have fruits with a more acidic flavour, rich in phenols with important health properties [6].

In recent years, there has been an increase in pomegranate fruit production. Pomegranate berries are considered a functional product of great benefit for the human diet due to the high nutraceutical content present in their juice. Hydrolysable tannins, anthocyanins and minerals contained in them have useful health properties [2], such as cancer prevention and therapy [7] and on chronic inflammatory diseases and cardiovascular diseases [8]. The beneficial effects and the antioxidant activity of the juice and fruit, in general, are attributed to the phenolic content that acts against free radicals, molecules highly harmful to the cells themselves. Moreover, today, the market demand comes from not only consumers but also from many foods and nutraceutical and cosmetics companies, and, for this reason, it has become increasingly important to characterize its different varieties to obtain high-quality products with economic interests.

Moreover, in the last few years in Italy, especially in the southern regions, there has been a rapid expansion of the areas cultivated with pomegranate that increased from 7 ha in 2008 to 1303 ha in 2020 [9]. In Sicily, pomegranate represents a minor fruit tree species even if it was cultivated since Arab domination, but it maintained great importance in private gardens and orchards until recent times. Thanks to uncontrolled sexual propagation that occurred from its introduction, nowadays, many local genotypes can be found in Sicily, likely well adapted to different climate conditions from the sea up to 800 m above sea level [10]. Recent studies were aimed to unveil the mechanism for pomegranate flavonoids production and accumulation [11,12,13]. At the same time, several investigations were carried out on genetic variability screening through molecular markers, i.e., simple-sequence repeat (SSR), allowing varietal identification [14,15,16,17,18]. However, all these studies relied only on neutral genes, which are not linked to market valuable traits. Therefore, the development of non-neutral markers associated with relevant traits can be highly useful for the rapid identification of new potential valuable varieties and future breeding programs. To make the most of the potential beneficial effects of new pomegranate genotypes, it is necessary to carry out wide-ranging research work of characterizing the various ecotypes.

Many studies have been published on the evaluation of the pomegranate germplasm using morphological, chemical and genetic variability, but an integrative approach integrating all these data would be beneficial for cultivar selection and improvement. In this study, an integrated morphological, chemical and molecular approach was used to characterize new pomegranate genotypes focusing on relevant nutraceutical traits, such as minerals and phenolic content.

## 2. Results and Discussion

### 2.1. Morphological Characteristics of Fruits

Morphological characteristics play an important role in consumer and market choice. Fruit size, aril yield and aril size are key traits for the fresh market and breeding programs. In this work, significant morphometric differences among the studied genotypes were found. The average fruit weight found in this study was 413.96 g, with high variability among genotypes, ranging from 255.87 g in G4 to a maximum of 825.82 g observed in WD (Table 1). Variation in fruit weight could depend on the cultivar and ecological condition [3]. These data showed that, as regards VL and WD, the average fruit weight was greater than reported in the literature [10,19], while the data of PS and DC were lower than reported by La Malfa et al. [10].

Moreover, the fruit circumference showed significant differences among genotypes, whose average was 303.35 mm. WD and G5 showed the highest values (386 mm and 362 mm, respectively), while the lowest value was observed in G4 (263 mm). Accordingly, WD and G5 displayed also the highest fruit length and diameter values, while the lowest values were found in G4 and PS, as shown in Table 1. The average fruit size of WD grown in Sicily was greater than the WD cultivated in California, as previously reported [19]. This could probably be due to the different climate, temperature and humidity conditions in the two environments. According to the fruit weight, WD also showed the highest weight of peel and arils (430.34 g and 395.48 g, respectively) (Table 1 and Table 2).

The latter result was in agreement with the value reported for the WD cultivar grown in Spain [20] but substantially differs from what was obtained for the same cultivar grown in America [19], probably, once again, due to dissimilar environmental growth conditions. The value reported for the arils weight of our VL (217.45 g) was lower than reported by Alcaraz-Mármol [20].

Because the ready-to-eat arils are attracting increasing international demand due to their health and nutraceutical characteristics, the arils’ features determine the economic value of the fruit and are important parameters for growers, market and industry [21]. The genotypes studied showed a large variability on arils traits: DC showed the highest arils yield (64.52%), while the lowest was observed in WD (47.77%), but, also, two Sicilian genotypes, G1 and G3, displayed high arils yield (62.48% and 62.95%, respectively). Furthermore, the G5 genotype has been shown to have the biggest arils, with a weight of 100 arils equal to 50.87 g; PS and WD have been shown to have the lowest (38.49 g and 38.60 g, respectively).

Moreover, the peel thickness is one important trait of market selection; fruits with thin skin and, therefore, with a lower peel yield, are more suitable for processing, while those with a thicker skin and higher peel yield are more suitable for transport and storage and can, therefore, be used for fresh consumption [3]. Regarding the peel, contrary to what was observed for arils yield, the WD genotype showed the highest peel yield (52.24%), while DC was the lowest (35.49%), showing that arils and peels yields are inversely correlated. However, the dry arils yield was highest in WD (23.08%), while dry peel yield was found to be highest in DC (36.05%), indicating that the percentage of humidity in these fruits, for the parameters analysed, is lower than the other cultivars examined.

### 2.2. Juice Characteristics

The juice yield is a very important trait from an industrial point of view to obtain bottled juice. According to the highest aril yield, DC also showed the highest juice yield, with 48.50% (Table 3); conversely, G5 and WD showed the lower value, with 33.18% and 33.58%, respectively. In general, the G1–G5 genotypes showed values of yield in arils and in juice higher or similar compared to the international genotypes (VL and WD). This classifies G1–G5 accessions as good products for the market and juice production.

Determination of total soluble solids (TSS or °Brix) is important to establish the organoleptic quality of the juice. The range of °Brix values found was from 15.76 in G2 to 17.75 in WD; all the varieties tested had a °Brix value higher than the minimum threshold generally required for commercial use (>12%). Our values are similar to those found in previous studies on different Apulian (from 13.60 to 18.00), Spanish (from 15.10 to 17.70) and Californian (from 14.90 to 16.80) pomegranate genotypes [17,19,20]. The value obtained for WD (17.75) was higher than that reported by the same previous studies (16.90, 17.20 and 16.80), but this may be due to environmental conditions and harvesting time [17,19,20].

The highest pH value was measured for G2, G3 and PS (3.76, 3.77 and 3.72, respectively), while the lowest was for G5 (3.15) and WD (3.14). These pH values were in agreement with Ferrara [17] and Beaulieu [22] for WD (2.93 and 3.05) and with Todaro [23] for PS, DC and VL (3.67, 3.88 and 3.71, respectively), growth in the experimental farm of the Catania University (Italy, Sicily), but they were lower than reported by Alcaraz-Mármol [20] for WD (3.89) and VL (5.90).

Observed as well were differences in juice colour among the genotypes (Table 3); G3 showed the highest values of lightness of colour (L) (22.40), whereas WD showed the lowest (13.47, 13.59 and 14.63, respectively). The highest values of colour a (tending to red colour) were observed in G4 (10.93), while G3 (4.49) and G5 (4.36) showed the greatest value of colour b, which means that the juice colour tends to be yellow. As regards value C, it was observed that G3, G4 and G5 were the genotypes with the most intensity/purity of the colour, with values of 11.21, 11.51 and 11.40, respectively. In addition, for the value of h°, significant differences were found between cultivars; a very high value was found in G3 (25.06). According to the CIELAB colour parameters, cultivars with values of colour, a positive and colour b negative have pigmentation from red to blue (G1, G2, PS, DC and VL), while cultivars with values of colour a and colour b positives have pigmentation from red to yellow (G3, G4, G5, and WD). Moreover, VL showed the lowest value of colour a, C and h°, which means that its juice tends towards a clear pink colour.

### 2.3. Total Phenolic Content and Antioxidant Activity

**Figure 1:**
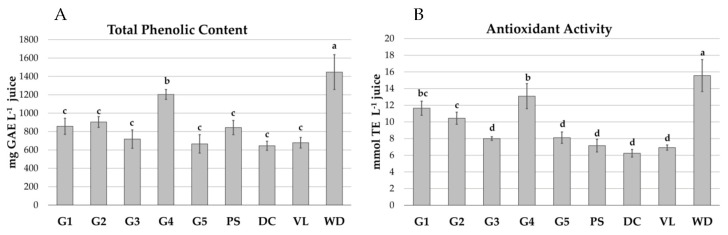
Total phenolic content (**A**) and antioxidant activity (**B**) in pomegranate juices. Each value is the mean of three replicates and is reported with standard deviation. Value bars indicated with different letters (from a to d) are significantly different (*p* ≤ 0.05).

Total phenolic content (TPC), expressed as gallic acid equivalent (GAE), ranged from 645.59 (in DC) to 1447.22 mg L^−1^ (in WD) (Figure 1A). The average value of TPC was 884.83 mg L^−1^, showing to be significantly influenced by the genotype (*p* ≤ 0.05). In particular, among the tested genotypes, the G4 showed the highest phenolic content (1203.77 mg L^−1^), comparable to the WD (1447.22 mg L^−1^), which is the most studied international genotype. Moreover, the genotypes with lower values were DC, G5 and VL. The values obtained were similar to those reported by Fanali [24] for the six old Italian pomegranate varieties collected in the experimental farm of Tuscia University (range from 500 to 1400 mg GAE L^−1^). In comparison with previous literature data, Sicilian genotypes showed TPC lower than some Croatian [25] (from 1985.6 to 2948.7 mg GAE L^−1^) and Iranian cultivars et al. [26] (from 2957.9 to 9853.2 mg GAE L^−1^).

The antioxidant activity (AA), expressed as Trolox equivalent (TE), followed the same trend of the TPC, with the mean value of 9.69 mmol L^−1^ varying significantly (*p* ≤ 0.05) between 6.24 in DC and 15.56 in WD (Figure 1B). WD showed the highest AA, followed by G4. The WD and VL values in this study were similar to those reported by Mena [27] (15.30 and 7.01, respectively) for the same cultivars grown in Spain (commercially available accessions).

The AA and TPC levels were positively and significantly correlated (*r* = 0.974).

### 2.4. Quantitative Determination of Carbohydrates by HPAE-PAD

The main carbohydrates detected in juices were glucose and fructose (Table 4).

The glucose concentration was mainly greater than fructose, with the ratio glucose/fructose taking values from 0.93 in G2 to 1.38 in DC. Similar profiles were previously described for other cultivars [28,29] (range from 0.96 to 1.12). In contrast, it is reported that the Spanish genotypes have almost always higher levels of fructose (from 55.4 to 82.4 g L^−1^) than glucose (from 55.3 to 78.0 g L^−1^) [30], as reported for WD (from 77.8 to 95.6 g L^−1^ for fructose and from 55.4 to 89.4 g L^−1^ for glucose) and VL (from 75.8 to 97.4 g L^−1^ for fructose and from 44.4 to 81.1 g L^−1^ for glucose) grown in Spain [20,27].

**Figure 2:**
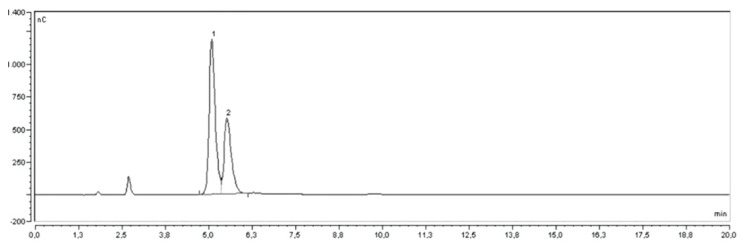
HPAE-PAD chromatogram of pomegranate juice (DC). Glucose (1) and fructose (2).

In this work, G2 showed the highest carbohydrate content, followed by WD. In particular, the highest amount of glucose was detected in G2 and in WD with 64.16 g L^−1^ and 62.54 g L^−1^, respectively, the lowest in G5 with 36.83 g L^−1^. A similar trend existed for fructose with G2, which showed also the highest amount of fructose (68.90 g L^−1^), while G5 was the lowest (27.84 g L^−1^). The sugar profile contributes to potential health benefits and determines the sensory attributes of pomegranate; juice red pomegranate cultivars usually have a sourer taste than pink-white genotypes. Figure 2 shows the chromatogram of one of the PJs (DC).

### 2.5. Quantitative Determination of Minerals by IC

The minerals in pomegranate juice, shown in Table 5, varied significantly among the genotypes. The highest content of macro-elements present in all the samples of juices was potassium (1816.73 mg L^−1^), followed by chlorides (416.90 mg L^−1^) and phosphates (367.04 mg L^−1^), according to Al-Maiman and Ahmad [31], who reported potassium as the highest among the mineral elements in pomegranate juice.

**Figure 3:**
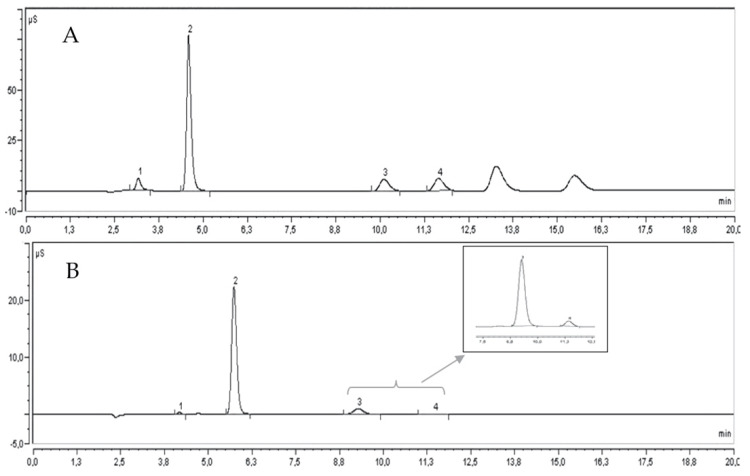
IC chromatogram of pomegranate juice (DC). Fluorides (1), chlorides (2), phosphates (3) and sulphates (4) (**A**). Sodium (1), potassium (2), magnesium (3) and calcium (4) (**B**).

Concerning anions (Figure 3A), the highest values were found in the genotype DC (1367.6 mg L^−1^), followed by WD (1234.20 mg L^−1^). Lower values were generally found in the Sicilian genotypes, especially in G1 (740.47 mg L^−1^). The relative order of concentration of anions was Cl^−^ > PO_4_^3−^ > SO_4_^2−^ > F^−^. The content of chlorides, sulphates and fluorides was higher in DC, whereas the content of phosphates was higher in WD.

Regarding cations (Figure 3B), the greatest results were generally found in WD (2350.17 mg L^−1^) and DC (2241.74 mg L^−1^); the lowest value was in G4 (1362.84 mg L^−1^). The order of concentration of cations was K^+^ > Mg^2+^ > Na^+^ > Ca^2+^, similar to that found by Al-Maiman and Ahmad [31] in ‘Taifi’ cultivars, except for the magnesium, which was lower. Potassium, sodium and calcium were predominant in WD, whereas magnesium was in G3. Overall, among the genotypes analysed, DC and WD showed the highest concentration of minerals, 3609.34 and 3584.37 mg L^−1^, respectively. The pomegranate juice appears to be a good source of nutrients, and variation in mineral composition could originate from the pomegranate genotypes as well as agro-climatic conditions, handling practices and manufacturing conditions.

### 2.6. Molecular Analyses

**Figure 4:**
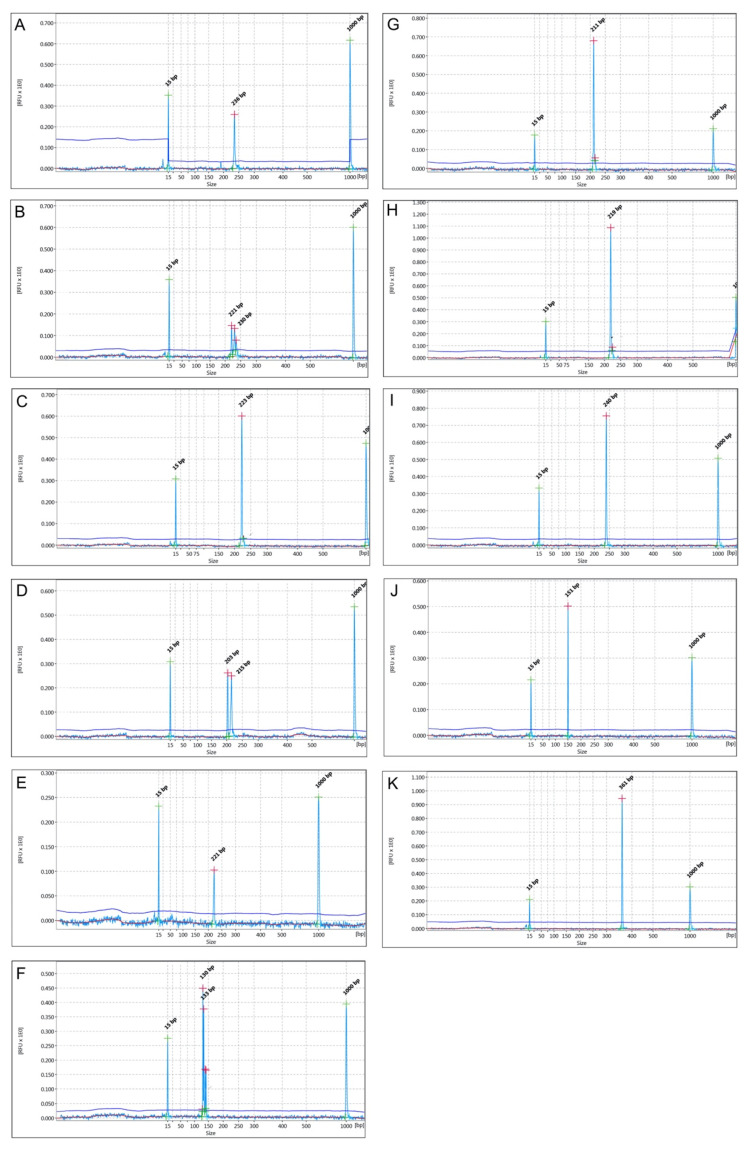
Capillary electrophoresis run of the PCR amplification products of ‘Wonderful’ (WD) variety with literature derived ones Pg4 (**A**), Pg10 (**B**), Pg14 (**C**), Pg21 (**D**), Pg22 I (**E**), Pg17 (**F**), Pom047 (**G**) and new developed markers MYBmp01 (**H**), MYBmp02 (**I**), MYBmp03 (**J**), MYBmp04 (**K**) and MYB derived markers were always homozygous, while Pg17, Pg21 and Pg10 were heterozygous. Peaks at 15 and 1000 bp correspond to the alignment marker used in the analysis.

Molecular analyses performed using SSRs produced a total of 96 alleles, with the maximum number of alleles per locus ranging from four to fourteen and a length of the amplified bands between 130 bp and 367 bp (Figure 4).

The polymorphism information content (PIC) was used to measure genetic diversity. High, medium or low loci polymorphism is in accordance with PIC > 0.5, 0.5 > PIC > 0.25 and PIC < 0.25, respectively [32]. A high PIC for all the markers used in this study was observed, on average 0.753, ranging from 0.469 of pg4 to 0.891 of pg14 (Table 6), higher than the previous analysis with the same microsatellites [32,33]. Among the new microsatellite markers developed in this study, MYBmp04 and MYBmp01 displayed a very high variability among the studied genotypes, with PIC values of 0.875 and 0.775, respectively (Table 6).

Although to a lesser degree, MYBmp02 and MYBmp03 markers showed relevant variability and all of them could be recommended for further genetic analyses aimed at detecting molecular diversity in the pomegranate germplasm collection. The Shannon index (I) and heterozygosity (He) values were consistent with the PIC trend. The Nei’s genetic diversity and Shannon’s information index in Sicilian pomegranates in the present study were higher in comparison to Indian pomegranates based on ISSR markers reported by Narzary [34]. It can be shown that the SSR marker is a trustworthy technique for assessing genetic diversity in pomegranate genotypes. The average number of alleles was 8.727, which is significantly higher with respect to what was reported for Iranian pomegranate genotypes analysed with chloroplast SSRs [35] and even with respect to previous analyses with nuclear microsatellites as well [33,36]. This set of four new MYB-related SSRs, along with the other seven SSRs, might be useful for population genetic analyses, such as genotyping and linkage mapping.

**Figure 5:**
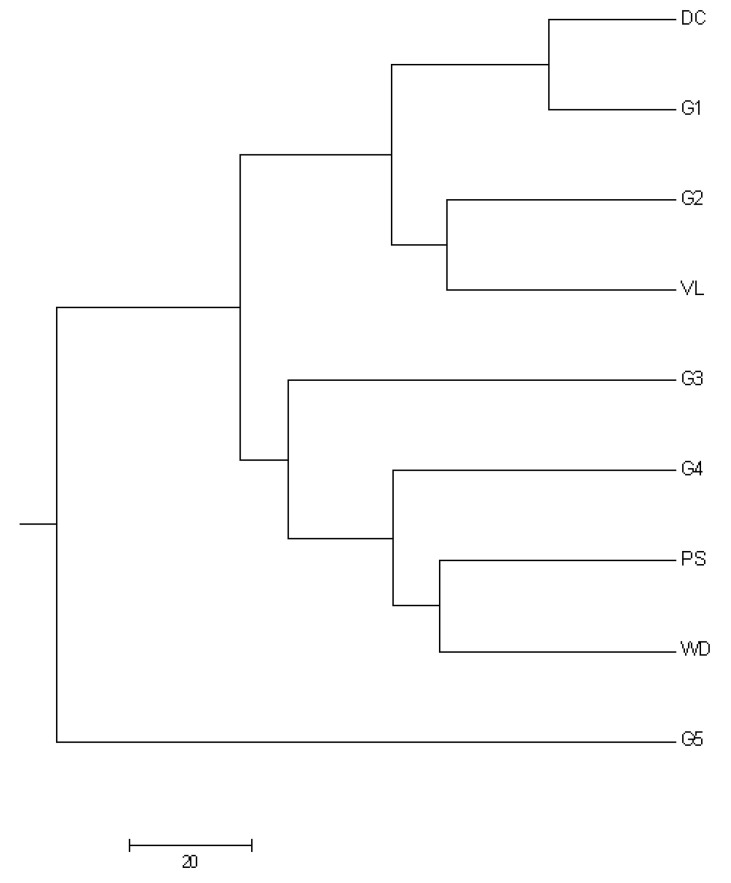
UPGMA dendrogram of 9 pomegranate genotypes based on the nuclear microsatellite (SSR) markers described in Table 6.

The cluster UPGMA (unweighted pair group method with arithmetic mean) analysis (Figure 5) performed with all 11 microsatellites clustered the tested genotypes into three main groups and showed that there is a genetic differentiation among the international genotypes.

The first one was composed of DC, G1, G2 and VL, while the second one included G3, G4, PS and WD. On the other hand, G5 was distinctly separated, forming an outgroup, from all the other tested varieties. DC and G1 clustered together; also, G4 and G3, WD and PS, in general, are closely related, while VL showed association with G1–G5 genotypes, in particular with G1, G2 and DC. The affinity between VL and most of the G1–G5 genotypes could be due to a common genetic origin since they have been domesticated within the Mediterranean basin; similarly, the clustering of PS together with WD could be explained by a common genetic matrix between the two, as shown by Parvaresh [32], referring to the “Palermo” genotype. This different clusterisation of the two international commercial varieties could have arisen from the highly different phenolic compounds composition between WD and VL, as reported along with this study.

### 2.7. Principal Component Analysis

**Figure 6:**
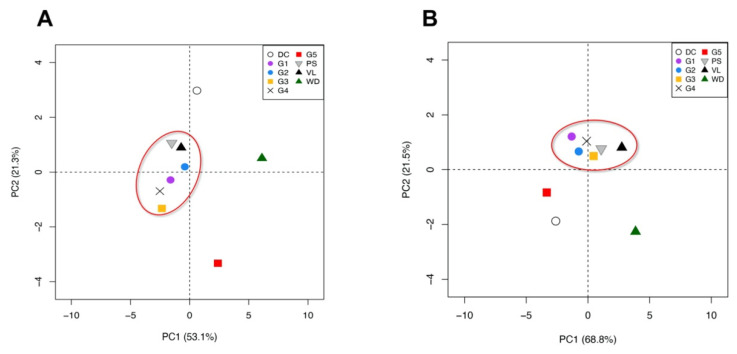
Principal component analysis of the sampled genotypes using morphological and physico-chemical characters (**A**) and four non-neutral SSRs (**B**). Represented values are the mean of 5 individual measurements.

The selection of the most valuable fruit characteristics, including fruit, aril and juice weight, shape (circumference), juice °Brix, juice pH, juice colour, minerals (as cations and anions) and antioxidant activity, allowed to identify a specific differentiation pattern in which G1–G4, ‘Valenciana’ and ‘Primosole’ are closely related, while DC, G5 and WD were apart from the group and between each other (Figure 6A).

The total variability of morphological and chemical factors is described by four factors, with the first two principal factors explaining 74.4%. The first factor accounted for 53.1%, while the second one contributed 21.3%. The PCA on molecular data (Figure 6B) revealed the same clustering pattern to the morpho-chemical one; in fact, the G1–G4 genotypes were grouped and G5, WD and DC were more isolated. The total genetic variability is described by four factors, with the first two principal factors explaining 90.3%. Differently from the result of UPGMA elaboration, the use of microsatellites derived from MYB related genes allowed a more efficient separation of genotypes reproducing the morpho-chemical characteristics. The Mantel test showed that flavonoid-associated SSR Euclidean distances were significantly correlated with morpho-chemical traits among the sampled genotypes (*r* = 0.62, *p* < 0.05). This finding could represent a valid tool to rapidly analyse the technological characteristics, such as fruit shape and weight, minerals content, juice features and antioxidant properties, of new pomegranate genotypes using genetic markers.

## 3. Materials and Methods

### 3.1. Plant Material

**Figure 7:**
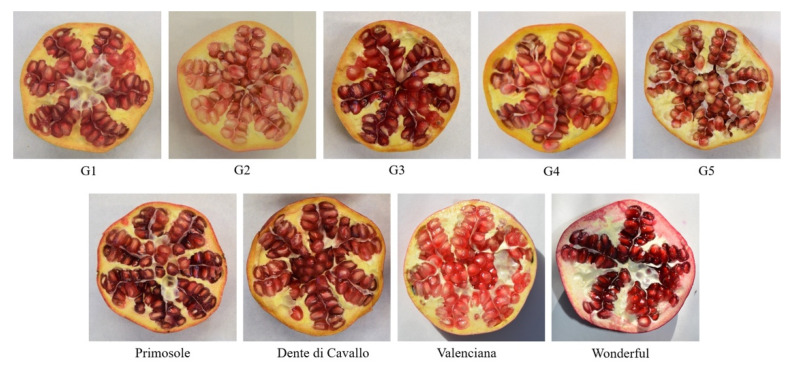
Cross section of pomegranate genotypes studied in this work.

The sampling was carried out on fruits and leaves of five pomegranate Sicilian genotypes, from ‘Genotype 1′ to ‘Genotype 5′ (from G1 to G5), two Sicilian commercial cultivars ‘Primosole’(PS) and ‘Dente di Cavallo’ (DC) and two international world-leading commercial varieties ‘Valenciana’(VL) and ‘Wonderful’(WD), collected in Sicily in October 2016 (Figure 7). All cultivars were harvested from a germplasm collection of CNR-ISAFOM, the section of Catania (Italy), grown under the same environmental conditions and with the same applied agronomic practices. For each accession, five fruits at the marketing ripeness stage were randomly collected.

### 3.2. Chemicals

Folin-Ciocalteau, sodium carbonate, 2,2-diphenyl-1-picrylhydrazyl (DPPH), methanol, 6-hydroxy-2,5,7,8-tetramethylchroman-2-carboxylic acid (Trolox), fructose, glucose, sodium hydroxide 50% and standard solutions for IC 1000 mg L^−1^ of sodium, potassium, magnesium, calcium, fluoride, chloride, sulphate and phosphate were purchased from Sigma-Aldrich (St. Louis, MO, USA). Methanesulphonic acid, sodium carbonate 0.5 M and sodium bicarbonate 0.5 M were obtained from Thermo Scientific (Waltham, MA, USA). Gallic acid was provided by Extrasynthese (Genay, France). Water Type I reagent grade was produced using a Milli-Q water purification system (Millipore, MA, USA).

### 3.3. Bio-Agronomic Traits and Physico-Chemical Analysis

Five mature fruits per genotype were analysed. Arils were manually separated from the peel and morphometric measurements were carried out on different fruit organs: peel, arils and seeds. Eighteen quantitative fruit morphological traits were examined, including fruit weight (g), fruit diameter (mm), fruit length (mm), fruit circumference (mm), number of septa, number of arils per fruit, arils weight (g), the weight of 100 arils per fruit (g), seeds weight (g), peel weight (g), juice weight (g). From these data, the peel yield (%), arils yield (%), juice yield (%) and seeds yield (%) were calculated. Furthermore, peel and arils were dried for 48 h at 105 °C and dry peel and arils yield (%) were determined. The pomegranate juice (PJ) was obtained by manual squeezing of arils through sterile gauze. The PJ was separated into aliquots and some juice was immediately frozen at −20 °C for downstream analyses. On the fresh juice, the following analyses were carried out: total soluble solids (TSS), pH and colour. The TSS was determined with a digital refractometer DBR 45 (Giorgio Bormac S.r.l., Carpi, Italy) and results were reported as °Brix at 20 °C. The pH measurements were performed using a digital pH meter XS Instruments mod. PC510 at 20 °C. The colour was measured with a Chroma Meter CR-400 (Konica Minolta, Osaka, Japan) using the CIELAB colour system [37]. Three colour measurements were made (L, a, b) and chroma (C) and hue (h°) units of colour space were detected.

### 3.4. Total Phenolic Content

TPC was determined by the method of Dewanto [38], with some modifications. The PJ, appropriately diluted with ultrapure water, was centrifuged at 5000 rpm for 4 min and 125 μL of supernatant was mixed with 625 μL of Folin–Ciocalteu reagent 5-fold-diluted with ultrapure water. After 6 min, 1.25 mL of 7% Na_2_CO_3_ aqueous solution and 1 mL of ultrapure water were added. The mixture was shaken and placed in the dark at room temperature for 1 h. After incubation, the total content of phenolic compounds was measured at 760 nm using the BioSpectrometer UV/Vis spectrophotometer (Eppendorf, Hamburg, Germany). Gallic acid standard solution (25–200 mg L^−1^) was used for the calibration curve (R^2^ = 0.9943). All measurements were performed in triplicate. The results were expressed as mg of gallic acid equivalent per litre of juice (mg GAE L^−1^).

### 3.5. Antioxidant Activity

AA of PJ was determined using the DPPH method described by Brand-Williams [39] with some modifications. PJ diluted in the ratio of 1:100 with methanol was centrifuged at 5000 rpm for 4 min. Afterwards, 100 µL of supernatant was mixed with 2 mL of 0.1 mM DPPH in methanol. After incubating at room temperature for 30 min in the dark, the absorbance of the mixture was measured at 517 nm. Trolox was used as a reference (calibration range 10–200 μmol L^−1^; R^2^ = 0.9992). All samples were analysed in triplicates and the results are expressed as mmol Trolox per litre of juice (mmol TE L^−1^).

### 3.6. Quantitative Determination of Carbohydrates by HPAE-PAD

The quantification of the main carbohydrates (fructose and glucose) present in PJ was determined by the method of Corradini [40]. PJ, diluted in the ratio 1:1000 with deionized water and filtered (0.45 µm) was analysed using a High-Performance Anion-Exchange chromatography with Pulsed Amperometric Detection (HPAE-PAD), Thermo Scientific Dionex ICS3000 (Sunnyvale, CA, USA), consisting of a quaternary gradient inert pump, a pulsed amperometric detector and AS40 automated sampler. The separation was carried out on a Dionex CarboPac PA10 analytical column (250 × 4 mm). The acquisition of all the chromatograms was performed with Chromeleon Chromatography Management System. All experiments were carried out at 30 °C under isocratic elution using NaOH 100 mM with a flow rate of 0.8 mL min^−1^. All analyses were performed in triplicate for each agronomic sample, quantified by calibration curve (range 0.5–100 mg L^−1^; R^2^ = 0.9982 for glucose and R^2^ = 0.9995 for fructose) and the results are reported in mg L^−1^. Relative standard deviations (RSD %) of peak retention times were <0.8%.

### 3.7. Quantitative Determination of Minerals by IC

The mineral content was determined using the ion chromatography (IC) method [41]. The most important inorganic anionic and cationic constituents of PJ were analysed: F^−^, Cl^−^, PO_4_^3−^ and SO_4_^2−^ for anions, Na^+^, K^+^, Mg^2+^ and Ca^2+^ for cations.

PJ was diluted in the ratio 1:100 with deionized water, filtered with a syringe filter 0.45 µm and subjected to analysis. All chromatographic analyses were performed by a Thermo Scientific Dionex ICS3000 ion chromatography (Sunnyvale, CA, USA) composed of an isocratic pump, a cationic or anionic suppressor, a conductance detector equipped with a temperature compensated conductivity cell, an injection valve with a 25 μL loop and a column thermostat compartment. The ion separation was carried out with two ion-exchange columns: anions were separated on a Dionex IonPac AS22 column (250 × 4 mm) with an IonPac AG22 guard column (50 × 4 mm) and cations were determined using an IonPac CS12A column (250 × 4 mm) equipped with IonPac CG12A guard column (50 × 4 mm).

An aqueous solution containing 20 mM methanesulfonic acid was used for the elution of cations. The mobile phase containing 4.5 mM sodium carbonate and 1.4 mM sodium bicarbonate was used for the elution of anions. Flow rate of 1.0 mL min^−1^ and 1.2 mL min^−1^ was used for the separation of cations and anions, respectively, maintaining the column temperature at 30 °C during analysis. All analyses were performed in triplicate for each agronomic sample, quantified by calibration curve (range 0.5–100 mg L^−1^; R^2^ > 0.999) and the results are reported in mg L^−1^ of juice. Relative standard deviations (RSD %) of peak retention times ranged from 0.7% to 2.1%.

### 3.8. Molecular Analyses

Genomic DNA was extracted from 100 mg of leaf pomegranate using the CTAB method described by Ebrahimi [33]. Samples quantity and quality were assessed by checking them on a 1% agarose gel and by measuring with an Eppendorf BioSpectrometer^®^ (Eppendorf AG, Hamburg, Germany) their absorbance at 260 nm and 280 nm to assess DNA purity.

Sixty-four loci characterized in previous studies [16,32,33,42,43,44] were considered for the analysis of genetic relationships among cultivars. Out of 64 loci, 7 markers were selected based on heterozygosity, the number of alleles, allele size and amplification reproducibility, preferring tetra- and tri-nucleotides compared to di-nucleotides. Furthermore, 4 new gene-derived primer pairs were designed using putative *Punica granatum* MYB gene sequences (GenBank Acc. num. HM056531.1 and MT495437.1) involved in the biosynthesis of flavonoids, as reported in Arlotta [12] (Table 7).

DNA was diluted to 30 ng μL^−1^ for PCR amplification. PCR assays were performed in a reaction mixture of 25 μL^−1^ including: 90 ng of genomic DNA, 0.25 μM of each primer, 200 μM dNTPs, 1 U of Q5 High-Fidelity DNA polymerase (New England Biolabs, Ipswich, MA, USA) and 4 μL^−1^ of 5x Q5 Reaction Buffer. DNA amplifications were performed in a thermocycler with the following cycling program: an initial denaturation step of 98 °C for 30 s, followed by 30 cycles of 98 °C for 10 s, at optimal annealing temperature for 30 s, 72 °C for 30 s and a final extension at 72 °C for 2 min. The amplification results were analysed by capillary electrophoresis using QIAxcel High Resolution Gel Cartridge (Qiagen, Hilden, Germany).

GeneALEx version 6.5 [45] was used to obtain a pairwise population matrix calculated through Nei’s genetic diversity, while expected heterozygosity (He), observed heterozygosity (Ho), Shannon index (I), the number of alleles and UPGMA dendrogram were calculated along with the number of alleles, major allele frequency, number of genotypes and PIC value for each primer.

### 3.9. Statistical Analysis

All analyses were carried out in triplicate and data were expressed as mean ± standard deviation. Pearson correlation coefficient (*r*) in bivariate linear correlation followed by Student’s test was used to compare phenolic content and antioxidant activity. Differences between means at the 95% (*p* ≤ 0.05) confidence level were considered statistically significant.

All data were submitted to Bartlett’s test for the homogeneity of variance and the data that were not homogeneous were logarithmically transformed. All the homogeneous data were analysed using analysis of variance (ANOVA) by CoSTAT program.

Two different principal component analyses (PCA) were performed to evaluate the potentiality of identified genetic markers. In the first PCA, we used morphological and chemical traits: arils, juice weight and fruit weight, circumference, juice °Brix, juice pH, colour L, chroma, TPC, AA, minerals (as cations and anions) and sugars. Another PCA was performed using only SSRs associated with flavonoid production. A Mantel test was performed to measure the correlation between the two matrices from each PCA as Euclidean distance matrices based on 9999 replications. Data were analysed using the R environment for statistical computing (R Development Core Team, 2021).

## 4. Conclusions

This study evaluated new pomegranate genotypes by a multidisciplinary approach using leading commercial varieties as a reference. A significant variability has been observed for the qualitative and chemical traits among the pomegranate genotypes. The SSR markers used in this study were suitable for the assessment of variability in the pomegranate germplasm for the preservation of this species. To the best of our knowledge, this research is the first study that described a common diversity pattern between the relevant bio-agronomic traits and genetic markers in *P. granatum*. The use of this set of *MYB*-related markers might represent a promising tool for the rapid genetic characterization of pomegranate accessions focused on marketable traits. Furthermore, all the microsatellite markers evaluated in the present study confirmed the relationships observed with morphological and chemical characteristics and can be used as genetic tools for breeding purposes.

As regards the nutraceutical content, the results showed that pomegranate juice is an excellent source of minerals that are essential for human health. Consuming a fruit per day, indeed, may cover the daily requirement of many minerals, especially potassium, most present in WD, DC and G5 among the genotypes investigated. Moreover, G4 presented the highest total phenolic content and antioxidant activity, even higher than the well-known marketable ‘Dente di Cavallo’, ‘Valenciana’ and ‘Primosole’. The G1–G3 and G5 genotypes presented a value of total phenolic content and antioxidant activity comparable to the commercial ones, representing a valid alternative to the most common cultivars for nutraceutical purposes.

## Figures and Tables

**Table 1:** Morphologic characteristics of fruit and peel.

Genotype	Fruit Weight (g)	Fruit Circumference (mm)	Fruit Length (mm)	Fruit Diameter (mm)	Fruit Shape (FL/FD)	Septum Number	Peel Weight (g)	Peel Yield (%)	Dry Peel Yield (%)
G1	285.72 ± 28.61 ^cd^	271.40 ± 8.65 ^e^	71.40 ± 2.71 ^d^	82.60 ± 2.41 ^de^	0.87 ± 0.02 ^a^	6.60 ± 0.55 ^b^	106.78 ± 10.39 ^d^	37.52 ± 3.53 ^c^	28.67 ± 3.49 ^cd^
G2	385.92 ± 29.97 ^c^	303.20 ± 10.24 ^c^	79.64 ± 1.68 ^c^	98.08 ± 8.55 ^c^	0.82 ± 0.07 ^a^	6.60 ± 0.55 ^b^	162.15 ± 17.11 ^c^	41.10 ± 2.22 ^bc^	27.53 ± 1.76 ^d^
G3	304.07 ± 34.41 ^cd^	276.00 ± 10.84 ^de^	74.40 ± 2.31 ^cd^	83.20 ± 2.78 ^de^	0.90 ± 0.03 ^a^	5.80 ± 0.45 ^b^	111.92 ± 11.63 ^d^	37.06 ± 4.74 ^c^	29.49 ± 1.87 ^cd^
G4	255.87 ± 23.26 ^d^	263.00 ± 7.88 ^e^	70.24 ± 2.57 ^d^	80.64 ± 3.74 ^e^	0.88 ± 0.04 ^a^	7.00 ± 1.00 ^b^	121.95 ± 22.29 ^d^	47.76 ± 8.64 ^ab^	33.02 ± 1.69 ^ab^
G5	632.42 ± 105.24 ^b^	362.00 ± 21.68 ^b^	90.74 ± 3.00 ^b^	106.44 ± 6.09 ^b^	0.86 ± 0.04 ^a^	8.20 ± 0.45 ^a^	295.72 ± 40.29 ^b^	47.27 ± 6.11 ^ab^	19.75 ± 1.21 ^e^
PS	297.85 ± 54.84 ^cd^	276.00 ± 16.74 ^cd^	67.60 ± 2.41 ^d^	82.40 ± 3.58 ^de^	0.83 ± 0.02 ^a^	6.20 ± 0.45 ^b^	117.74 ± 16.10 ^d^	40.07 ± 6.06 ^bc^	33.96 ± 2.38 ^ab^
DC	359.42 ± 46.66 ^cd^	295.00 ± 11.73 ^cd^	79.56 ± 5.49 ^c^	90.62 ± 4.65 ^d^	0.88 ± 0.07 ^a^	7.00 ± 0.00 ^b^	127.79 ± 23.83 ^d^	35.49 ± 4.41 ^c^	36.05 ± 0.97 ^a^
VL	378.51 ± 28.24 ^c^	297.60 ± 3.37 ^cd^	80.98 ± 6.05 ^c^	90.00 ± 2.47 ^d^	0.91 ± 0.08 ^a^	7.00 ± 0.00 ^b^	161.06 ± 24.53 ^c^	42.49 ± 4.97 ^bc^	31.28 ± 2.91 ^bc^
WD	825.83 ± 142.80 ^a^	386.00 ± 19.62 ^a^	105.05 ± 8.61 ^a^	114.65 ± 5.18 ^a^	0.92 ± 0.06 ^a^	6.75 ± 1.50 ^b^	430.34 ± 70.61 ^a^	52.24 ± 3.02 ^a^	20.97 ± 1.54 ^e^

Data are expressed as mean ± SD (*n* = 3). Significant differences (*p* ≤ 0.05) are indicated by different letters (from ^a^ to ^e^). FL, fruit length; FD, fruit diameter; PS, ‘Primosole’; DC, ‘Dente di Cavallo’; VL, ‘Valenciana’; WD, ‘Wonderful’.

**Table 2:** Morphologic characteristics of arils and seeds.

Genotype	Number of Arils	Arils Weight (g)	Weight of 100 Arils (g)	Arils Yield (%)	Dry Arils Yield (%)	Seeds Weight (g)	Seeds Yield (%)
G1	464 ± 79.80 ^bc^	178.94 ± 25.61 ^b^	39.98 ± 1.96 ^b^	62.48 ± 3.54 ^ab^	16.35 ± 0.83 ^d^	54.59 ± 10.80 ^c^	19.04 ± 2.58 ^ab^
G2	568 ± 50.23 ^bc^	223.78 ± 17.42 ^b^	43.00 ± 4.32 ^b^	58.02 ± 2.23 ^abcd^	16.73 ± 0.76 ^cd^	80.42 ± 3.89 ^b^	20.90 ± 1.21 ^a^
G3	479 ± 107.12 ^bc^	192.15 ± 31.89 ^b^	41.84 ± 3.46 ^b^	62.95 ± 4.74 ^ab^	17.17 ± 0.87 ^cd^	53.15 ± 3.78 ^c^	17.59 ± 1.58 ^abc^
G4	372 ± 87.48 ^c^	133.92 ± 26.77 ^c^	38.76 ± 4.3 ^b^	52.24 ± 8.64 ^bcd^	17.97 ± 0.26 ^bc^	42.68 ± 8.07 ^c^	16.59 ± 1.86 ^bc^
G5	674 ± 146.24 ^b^	336.70 ± 85.89 ^a^	50.87 ± 1.86 ^a^	52.73 ± 6.12 ^cd^	16.93 ± 0.56 ^cd^	123.63 ± 22.48 ^a^	19.54 ± 1.34 ^ab^
PS	510 ± 158.46 ^bc^	180.11 ± 45.77 ^b^	38.49 ± 5.93 ^b^	59.94 ± 6.06 ^abc^	18.99 ± 1.27 ^b^	46.29 ± 9.00 ^c^	15.66 ± 2.85 ^bc^
DC	550 ± 78.25 ^bc^	231.63 ± 31.62 ^b^	43.85 ± 2.74 ^b^	64.52 ± 4.41 ^a^	18.03 ± 0.45 ^bc^	57.32 ± 5.81 ^c^	16.02 ± 1.17 ^bc^
VL	543 ± 104.58 ^bc^	217.45 ± 22.10 ^b^	43.01 ± 6.53 ^b^	57.52 ± 4.97 ^abcd^	17.29 ± 0.74 ^cd^	50.60 ± 14.03 ^c^	13.44 ± 4.16 ^c^
WD	1028 ± 219.96 ^a^	395.48 ± 80.39 ^a^	38.60 ± 3.42 ^b^	47.77 ± 3.02 ^d^	23.08 ± 0.51 ^a^	118.51 ± 34.84 ^a^	14.19 ± 2.16 ^c^

Data are expressed as mean ± SD (*n* = 3). Significant differences (*p* ≤ 0.05) are indicated by different letters (from ^a^ to ^d^). PS, ‘Primosole’; DC, ‘Dente di Cavallo’; VL, ‘Valenciana’; WD, ‘Wonderful’.

**Table 3:** Physico-chemical characteristics and colour coordinates (L, a, b, C and h°) of pomegranate juice.

Genotype	Juice Weight (g)	Juice Yield (%)	pH	Total Soluble Solids (°Brix)	Lightness (L)	Colour a	Colour b	Chroma (C)	Hue (h°)
G1	124.34 ± 16.87 ^cd^	43.43 ± 2.47 ^abc^	3.63 ± 0.08 ^ab^	16.06 ± 0.19 ^bc^	17.70 ± 0.93 ^b^	6.84 ± 0.67 ^bc^	−1.62 ± 0.43 ^c^	7.05 ± 0.58 ^bc^	−13.51 ± 4.39 ^c^
G2	143.35 ± 15.72 ^cd^	37.12 ± 2.57 ^bcd^	3.76 ± 0.10 ^a^	15.76 ± 0.63 ^c^	17.77 ± 0.80 ^b^	5.86 ± 0.60 ^c^	−1.70 ± 0.19 ^c^	6.11 ± 0.58 ^bc^	−16.27 ± 2.19 ^c^
G3	139.00 ± 30.61 ^cd^	45.35 ± 6.10 ^ab^	3.77 ± 0.08 ^a^	16.06 ± 0.58 ^bc^	22.40 ± 4.05 ^a^	10.49 ± 3.42 ^a^	4.49 ± 1.47 ^a^	11.21 ± 3.02 ^a^	25.06 ± 8.27 ^a^
G4	91.23 ± 20.76 ^d^	35.64 ± 7.47 ^cd^	3.54 ± 0.06 ^b^	17.00 ± 0.37 ^ab^	18.69 ± 1.33 ^ab^	10.93 ± 1.47 ^a^	2.52 ± 3.52 ^ab^	11.51 ± 2.51 ^a^	10.82 ± 13.15 ^b^
G5	213.06 ± 64.13 ^b^	33.18 ± 5.34 ^d^	3.15 ± 0.05 ^c^	16.08 ± 0.51 ^bc^	21.06 ± 5.51 ^ab^	10.68 ± 3.37 ^a^	4.36 ± 1.94 ^a^	11.40 ± 4.07 ^a^	19.36 ± 7.84 ^ab^
PS	133.81 ± 40.00 ^cd^	44.27 ± 5.39 ^abc^	3.72 ± 0.07 ^a^	17.52 ± 0.85 ^a^	18.57 ± 0.27 ^ab^	8.75 ± 1.10 ^ab^	−1.33 ± 0.09 ^b^	8.81 ± 1.09 ^ab^	−8.60 ± 1.20 ^c^
DC	174.31 ± 26.58 ^bc^	48.50 ± 3.78 ^a^	3.64 ± 0.03 ^ab^	17.54 ± 0.21 ^a^	18.07 ± 1.78 ^b^	6.66 ± 1.03 ^bc^	−0.83 ± 1.01 ^c^	6.78 ± 0.95 ^bc^	−7.70 ± 9.23 ^c^
VL	166.85 ± 20.15 ^bc^	44.08 ± 4.11 ^abc^	3.67 ± 0.10 ^ab^	17.58 ± 0.60 ^a^	19.59 ± 1.50 ^ab^	4.88 ± 1.34 ^c^	−1.59 ± 0.64 ^c^	5.19 ± 1.19 ^c^	−19.30 ± 9.41 ^c^
WD	276.98 ± 51.18 ^a^	33.58 ± 2.57 ^d^	3.14 ± 0.20 ^c^	17.75 ± 0.86 ^a^	13.59 ± 0.74 ^c^	7.08 ± 1.67 ^bc^	1.30 ± 0.37 ^bc^	7.20 ± 1.71 ^bc^	10.32 ± 1.15 ^b^

Data are expressed as mean ± SD (*n* = 3). Significant differences (*p* ≤ 0.05) are indicated by different letters (from ^a^ to ^d^). PS, ‘Primosole’; DC, ‘Dente di Cavallo’; VL, ‘Valenciana’; WD, ‘Wonderful’.

**Table 4:** Carbohydrate content (g L^−1^) of pomegranate juices.

Genotype	Glucose (G)	Fructose (F)	Total	Ratio G/F
G1	45.33 ± 0.79 ^de^	33.54 ± 0.39 ^de^	78.87	1.35
G2	64.16 ± 0.09 ^a^	68.90 ± 0.24 ^a^	133.06	0.93
G3	43.18 ± 1.20 ^ef^	32.03 ± 0.80 ^e^	75.20	1.35
G4	46.21 ± 2.29 ^d^	34.44 ± 1.54 ^d^	80.65	1.34
G5	36.83 ± 0.96 ^g^	27.84 ± 0.63 ^g^	64.67	1.32
PS	48.69 ± 0.28 ^c^	35.51 ± 0.15 ^d^	84.20	1.37
DC	52.81 ± 1.16 ^b^	38.36 ± 1.36 ^c^	91.17	1.38
VL	40.96 ± 2.62 ^f^	29.89 ± 1.72 ^f^	70.85	1.37
WD	62.54 ± 0.23 ^a^	62.50 ± 0.04 ^b^	125.04	1.00

Data are expressed as mean ± SD (*n* = 3). Significant differences (*p* ≤ 0.05) are indicated by different letters (from ^a^ to ^g^). PS, ‘Primosole’; DC, ‘Dente di Cavallo’; VL, ‘Valenciana’; WD, ‘Wonderful’.

**Table 5:** Minerals (mg L^−1^) of pomegranate juices.

Parameter	G1	G2	G3	G4	G5	PS	DC	VL	WD	Mean
**Anions (A-)**										
Fluorides	42.97 ± 1.52 ^e^	41.80 ± 0.72 ^e^	48.27 ± 1.33 ^c^	45.60 ± 1.52 ^d^	41.67 ± 0.29 ^e^	39.03 ± 1.22 ^f^	59.90 ± 0.61 ^a^	42.37 ± 0.42 ^e^	51.33 ± 0.75 ^b^	**45.88**
Chlorides	365.17 ± 7.88 ^f^	296.07 ± 2.02 ^h^	317.63 ± 2.87 ^g^	380.83 ± 3.86 ^e^	285.70 ± 1.04 ^h^	489.63 ± 7.94 ^c^	637.10 ± 12.00 ^a^	422.00 ± 1.67 ^d^	557.97 ± 6.33 ^b^	**416.90**
Phosphates	262.80 ± 5.95 ^h^	333.40 ± 3.95 ^d^	310.87 ± 7.49 ^f^	294.47 ± 4.91 ^g^	317.17 ± 2.72 ^e^	387.67 ± 0.61 ^c^	463.87 ± 8.28 ^b^	387.80 ± 0.36 ^c^	545.30 ± 8.31 ^a^	**367.04**
Sulphates	69.53 ± 0.40 ^i^	83.70 ± 1.51 ^g^	113.20 ± 1.45 ^d^	90.93 ± 2.01 ^f^	139.97 ± 1.40 ^b^	126.60 ± 0.44 ^c^	206.73 ± 3.23 ^a^	93.97 ± 1.21 ^e^	79.60 ± 1.47 ^h^	**111.58**
**Total A-**	**740.47**	**754.97**	**789.97**	**811.83**	**784.51**	**1042.93**	**1367.60**	**946.14**	**1234.20**	
**Cations (C+)**										
Sodium	9.21 ± 0.92 ^e^	13.33 ± 0.49 ^c^	13.58 ± 0.24 ^c^	12.81 ± 0.31 ^cd^	11.70 ± 1.05 ^d^	14.08 ± 0.14 ^c^	18.53 ± 1.69 ^a^	16.09 ± 0.88 ^b^	18.84 ± 0.86 ^a^	**14.24**
Potassium	1710.42 ± 19.95 ^d^	1819.33 ± 13.83 ^c^	1679.56 ± 68.23 ^d^	1287.99 ± 70.45 ^e^	2056.63 ± 19.41 ^b^	1668.04 ± 0.56 ^d^	2142.66 ± 16.43 ^b^	1749.37 ± 28.68 ^d^	2236.56 ± 51.49 ^a^	**1816.73**
Magnesium	54.32 ± 1.26 ^c^	57.57 ± 0.54 ^b^	72.08 ± 3.02 ^a^	53.98 ± 2.81 ^c^	69.58 ± 0.61 ^a^	49.00 ± 1.02 ^d^	70.60 ± 0.73 ^a^	48.75 ± 0.93 ^d^	57.84 ± 1.85 ^b^	**59.30**
Calcium	8.66 ± 1.69 ^c^	7.67 ± 1.06 ^c^	6.98 ± 0.40 ^c^	8.05 ± 0.69 ^c^	30.38 ± 1.20 ^b^	7.29 ± 0.53 ^c^	9.95 ± 1.18 ^c^	8.15 ± 1.60 ^c^	36.93 ± 1.20 ^a^	**13.78**
**Total C+**	**1782.61**	**1897.90**	**1772.20**	**1362.83**	**2168.29**	**1738.41**	**2241.74**	**1822.36**	**2350.17**	
**Total Minerals**	**2523.08**	**2652.87**	**2562.17**	**2174.66**	**2952.80**	**2781.34**	**3609.34**	**2768.50**	**3584.37**	

Data are expressed as mean ± SD (*n* = 3). Significant differences (*p* ≤ 0.05) are indicated by different letters (from ^a^ to ^i^). PS, ‘Primosole’; DC, ‘Dente di Cavallo’; VL, ‘Valenciana’; WD, ‘Wonderful’.

**Table 6:** Microsatellite allele data obtained using 11 polymorphic microsatellite loci in the tested pomegranate genotypes.

Locus	Major Allele Frequency	Genotypes Number	Alleles Number	Ho	He	Nei	I	PIC
Pg4	0.667	4	4	0.000	0.521	0.510	0.961	0.469
Pg10(a)	0.458	7	7	0.542	0.704	0.689	1.392	0.642
Pg14	0.167	14	14	0.000	0.918	0.899	2.463	0.891
Pg21	0.250	14	11	0.458	0.887	0.869	2.205	0.862
Pg22	0.250	10	10	0.000	0.876	0.858	2.109	0.843
Pg17	0.313	13	9	0.417	0.836	0.819	1.902	0.797
Pom047	0.208	10	10	0.000	0.894	0.875	2.178	0.862
MYBmp01	0.292	8	8	0.000	0.819	0.802	1.794	0.775
MYBmp02	0.458	7	7	0.000	0.723	0.708	1.516	0.671
MYBmp03	0.542	5	5	0.000	0.653	0.639	1.258	0.597
MYBmp04	0.208	11	11	0.000	0.904	0.885	2.279	0.875
**Average**	**0.347**	**9.364**	**8.727**	**0.129**	**0.794**	**0.778**	**1.823**	**0.753**

Ho, observed heterozygosity; He, expected heterozygosity; Nei, Nei’s genetic diversity; I, Shannon’s information index; PIC, polymorphism information content.

**Table 7:** Primer sets used to investigate genetic diversity among pomegranate cultivars and the annealing temperature (Ta) chosen for each primer.

Locus	Repeat Motif	Primer Sequence (5’-3’)	Ta °C	Reference
Pg4	(TC)12 TT(TC)20	F: CTGATGTAATGGCTGAGCAAA	63	Ebrahimi et al. 2010
		R: GCACTTGAACAAAGAGAATGC		
Pg10(a)	(AG)9 GG(AG)14	F: TGCTAGACAGAACTGGGAGAAC	63	Ebrahimi et al. 2010
		R: AGAGAGTGGGGTTTCCATTG		
Pg14	(AG)32	F: GCACATTTCTTCCACCTTCC	62	Ebrahimi et al. 2010
		R: GGTTACAATGCACAGAGTCCAC		
Pg21	(AG)7	F: CAAGACAGAAGCACCATCCA	62	Ebrahimi et al. 2010
		R: TCTCCCAAATCAGACCAACC		
Pg22	(ACAT)3 (AT)3 (AG)22 (AT)3	F: CCCCGCACTTAGAATCTATTA	56	Ebrahimi et al. 2010
		R: TCCAGTTCCAATCGACAGAC		
Pg17	(TCA)14	F: CATCAGACTACGATGGCACT	57	Parvaresh et al. 2010
		R: GCATAATAGCCTTCAATTTACA		
Pom047	(CT)24	F: GCCTATCTCGTGATCACATC	57	Rania et al. 2012
		R: AATGGGAGCGGACTAACTAT		
MYBmp01	(CT)9	F: GATGAAGATGACAAAACACCCC	60	Present study
		R: TGGGAGCTAGACAGAGTGACAA		
MYBmp02	(GA)12	F: TCCTCAAGCAGACCCAGAAA	62	Present study
		R: TGCTGTTCTTGTTACGCCTT		
MYBmp03	(AGC)4	F: AGGCGTAACAAGAACAGCAA	62	Present study
		R: AGCAACAGTCTTCCACCTCC		
MYBmp04	(GAG)4	F: CTCGCTTGTCTTGCTAAAGGAT	57	Present study
		R: CGAGGAACTTATTGACCCACTC		

## Data Availability

Not applicable.

## Sample Availability

Samples of the compounds are not available from the authors.
